# Supplementary material for: Assessment of pediatric surgical needs, health-seeking behaviors, and health systems in a rural district of Pakistan
Source: PLOS Glob Public Health. 2022 Dec 30;2(12):e0000810. doi: 10.1371/journal.pgph.0000810 (PMC10021200; doi:10.1371/journal.pgph.0000810)
Supplement: S1 Table — (DOCX) [file pgph.0000810.s001.docx]

**S1 Table: Diagnoses of photographs taken at the study site**

**Table A: Chest**

| **Region** | **Diagnosis of lesion** | **Number of lesions** |
| --- | --- | --- |
| Chest | Tetralogy of fallot | 1 |
|  | Ventricular Septal Defect | 3 |
|  | Pectus excavatum | 5 |
|  | Chest wall lymphangioma | 1 |
|  | Mitral regurgitation | 1 |
|  | Laryngomalacia | 1 |

**Table B: Abdomen**

| **Region** | **Diagnosis of lesion** | **Number of lesions** |
| --- | --- | --- |
| Abdomen | Umbilical hernia | 22 |
|  | Abdominal distension | 4 |
|  | Soft tissue tumor | 1 |
|  | Renal stone | 3 |
|  | Hemangioma | 2 |
|  | Bladder stone | 2 |
|  | Umbilical granuloma | 1 |
|  | Ileal atresia repair | 1 |

**Table C: Buttocks/Groin/Genitalia**

| **Region** | **Diagnosis of lesion** | **Number of lesions** |
| --- | --- | --- |
| Buttocks/ groin /genitalia | Inguinal hernia | 7 |
|  | Undescended testes | 2 |
|  | Hypospadias | 1 |
|  | Exstrophy bladder | 1 |
|  | Hydrocele | 1 |
|  | Adrenogenital syndrome | 1 |
|  | Gluteal trauma | 1 |
|  | Gluteal burn | 3 |
|  | Gluteal abscess | 1 |

**Table D: Back**

| **Regions** | **Diagnosis of lesion** | **Number of lesions** |
| --- | --- | --- |
| Back | Spina bifida | 1 |
|  | Soft tissue tumor | 2 |
|  | Hemangioma | 3 |
|  | Back burn | 2 |

**Table E: Extremities**

| **Region** | **Diagnosis of lesion** | **Number of lesions** |
| --- | --- | --- |
| Upper limb | Congenital upper limb anomaly | 3 |
|  | Nail bed infection | 1 |
|  | Polydactyly | 6 |
|  | Wound infection | 1 |
|  | Hand burn | 4 |
|  | Post-burn contracture | 2 |
|  | Clavicle fracture | 2 |
|  | Hand injury infection | 1 |
|  | Congenital thumb anomaly | 2 |
|  | Lipoma | 1 |
|  | Soft tissue infection | 1 |
| Lower limb | Femur fracture | 2 |
|  | Knock knee genu valgum | 3 |
|  | Lower limb anomaly | 2 |
|  | Club foot | 1 |
|  | Soft tissue infection | 3 |
|  | Pes Plano valgus | 5 |
|  | Arthrogryposis | 1 |
|  | Metatarsus adductus | 2 |
|  | Septic arthritis | 1 |
|  | Leg trauma | 3 |
|  | Ankle injury | 1 |
|  | Trauma abrasion | 2 |
|  | Dropped toe | 1 |
|  | Foot ganglion | 1 |
|  | Lower feet | 1 |
|  | Foot injury | 1 |
|  | Abrasion | 1 |
|  | Foot bony tumor | 1 |
|  | Planter warts | 1 |
|  | Extensor halluces longus injury | 1 |
|  | Lymphangitis lower limb | 1 |
|  | Juvenile planus | 1 |

**Table F: Head and Neck**

| **Region** | **Diagnosis of lesion** | **Number of lesions** |
| --- | --- | --- |
| Eyes | Squint | 26 |
|  | Ptosis | 3 |
|  | Anophthalmia | 7 |
|  | Eye infection | 1 |
|  | Microphthalmia | 2 |
| Ears | Discharging ear | 6 |
|  | Otitis externa | 5 |
|  | Ear Microtia | 2 |
|  | Fungal ear infection of pinna | 1 |
|  | Herpes infection of skin | 1 |
| Face | Cheek malformation | 5 |
|  | Hemangioma | 1 |
|  | Cleft lip | 6 |
|  | Eye brow laceration | 1 |
|  | Face injury | 2 |
|  | Pre-auricular sinus | 1 |
|  | Pre-auricular skin tag | 5 |
|  | Post-auricular skin infection | 1 |
|  | Parotid swelling | 1 |
|  | Nose trauma | 2 |
|  | Fascial burn | 1 |
|  | Tongue tie | 1 |
|  | Forehead trauma | 2 |
|  | Soft tissue infection | 2 |
|  | Angular dermoid | 1 |
| Neck | Torticollis | 1 |
|  | Hemangioma | 3 |
|  | Neck mass | 1 |
|  | Neck injury | 1 |
|  | Neck cyst | 2 |
|  | Skin infection | 1 |
| Head | Scalp infection | 1 |
|  | Scalp hemangioma | 2 |
|  | Encephalocele | 1 |
