# Supplementary material for: Assessment of pediatric surgical needs, health-seeking behaviors, and health systems in a rural district of Pakistan
Source: PLOS Glob Public Health. 2022 Dec 30;2(12):e0000810. doi: 10.1371/journal.pgph.0000810 (PMC10021200; doi:10.1371/journal.pgph.0000810)
Supplement: Attachment — Submitted filename: Response to reviewers.docx [file pgph.0000810.s002.docx]

**Responses to queries: Assessment of pediatric surgical needs, health-seeking behaviors, and health systems in the rural district of Tando Mohammad Khan Sindh, Pakistan, PGPH-D-22-01041**

| S. No |  |  |
| --- | --- | --- |
| 1 | Please amend your detailed online Financial Disclosure statement. This is published with the article. It must therefore be completed in full sentences and contain the exact wording you wish to be published. | All the authors have declared no financial interests. |
| 2 | Please state the initials, alongside each funding source, of each author to receive each grant. | Dr. Jai K. Das – Bill and Malinda Gates Foundation. |
| 3 | Please update your online Competing Interests statement. If you have no competing interests to declare, please state: “The authors have declared that no competing interests exist.” | The authors have declared that no competing interests exist. |
| 4 | We have noticed that you have uploaded Supporting Information files, but you have not included a list of legends. Please add a full list of legends for your Supporting Information files after the references list. | Added |
|  | Reviewer 1 |  |
|  |  | We would like to thank the reviewer for such a thorough review of the draft, which has really helped in shaping the draft and adding clarity |
| 5 | Title: I would consider saying “a rural district in Pakistan” rather than spelling out the district name. This would cut a lot of characters and simplify it a bit. Readers can see the specifics when they read the article, and the specific place is not needed in the title | Thanks, we have now edited to: Assessment of pediatric surgical needs, health-seeking behaviors, and health systems in a rural district of Pakistan |
|  | Abstract: |  |
| 6 | DALY please spell out on first mention with the acronym in parentheses. | Revised |
| 7 | On first mention of all acronyms, please spell out in full. Also spell out district name in full on first mention. | Revised |
| 8 | You mention the names of the survey tools but do not define them or describe what they are assessing. In the methods you need to describe these tools better, as not knowing in a general sense what they are measuring inhibits my ability to interpret the results section. | Added: The SOSAS survey tool is a structured questionnaire that helps identify the locations of injury, the cause of injury, the care sought, and if any long-term disabilities left by the injury are present.  Health facilities were assessed using the PediPIPES survey form, this form helps identify key structural components of a health care setup namely the staff and the facilities available to deal with medical conditions.  The details are in the manuscript. |
| 9 | No need to include ethical approval in the abstract. This can be in the body of the manuscript instead. | Edited and removed |
| 10 | Please spell out healthcare facilities in the district as I do not know what all the acronyms (like RHCs, BHUs, DCDs, etc) are. | Revised this portion and removed these details of health facilities categorization |
| 11 | In the methods you need at least a sentence about the statistical analysis, even if it was just saying that descriptive statistics were performed. | Thanks and we have now added these details.  Added: Descriptive analysis was performed using STATA 16 (Stata Statistical Software: Release 16. College Station, TX: StataCorp LP) |
| 12 | I would recommend better organization of the results section. Rather than listing the different areas of the body with frequencies, I would focus on the areas that you feel warrant mention the most, such as greatest number of lesions and most surgical need, as well as least. I would not list them all in a laundry list. | Thanks for this valuable suggestion.  We have now revised it accordingly. |
| 13 | I would like a bit more explanation on what “unmet surgical need” means. How are you measuring that? | Added: The current surgical need was defined as the mother’s / caregiver’s self-reported surgical problems in their children, either present at the time of the surveys or in the past. The unmet surgical need was defined as a surgical problem for which the respondent did not or could not access care. |
| 14 | The result section of the abstract that discusses the health facilities is currently pretty meaningless without the definitions of what the different facilities are. | Thanks, we have removed these unnecessary details. |
| 15 | What does the PediPIPES score mean? Just a brief description would go a long way here. | We have added this briefly in the abstract and details in the methods.  It was calculated by allocating 1 point for each personnel, infrastructure, procedures, equipment and supplies present in the facility. A total PediPIPES score is calculated by summing the number of the Personnel, Infrastructure items and number of operating rooms, Procedure, Equipment and Supplies scores. This number was then divided by the total number of data items (118) and multiplied by 10 to create the PediPIPES index. There is no maximum number for the PediPIPES index. It represents the overall capacity of the hospital to cater with a pediatric surgical case. |
| 16 | The first sentence of the conclusion is an overstatement. I think I would say it holds great significance for one area of Pakistan, or even just cut the first sentence and go straight into the second sentence, “This study provides important insight into the birden of children’s operative disease in Pakistan’s rural district of TMK.” | Revised: This study provides important insight into the burden of children’s operative disease in Pakistan’s rural district of TMK but this district would be representative of any rural district of Sindh has been shown in multiple surveys over the years with comparable results. |
|  | Introduction |  |
| 17 | Please write out LMICs at first mention and put acronym in parentheses (line 70).  p. In line 74 “reserved largely for those who can pay for them”, do you mean pay out of pocket or with insurance? Clarify here. | Thanks, we have spelled out all the acronyms the first time.  Revised: reserved largely for those who can pay for them out of pocket |
| 18 | . In line 84-85, you mention individual understanding of these disease pathologies. Do you mean patient understanding or healthcare provider understanding? Which of these are you measuring in this study? | Revised: local population’s understanding of these disease pathologies, |
| 19 | Line 87 I would say “as children OFTEN lack the ability to express their health issues”, as many children are able to express their health issues quite well. | Revised |
| 20 | The paragraph from lines 83-97 would benefit from a little bit of organization, as it goes from Health-related decisions handled by parents and families to surveys rather abruptly. Consider a transition here. | Revised |
| 21 | In line 104-105, the first sentence of the last paragraph is too long and disjointed. I would cut “keeping in mind the global trends of surgical care delivery depicted in past research” and keep the rest the same. | Revised |
| 22 | Line 109 “form” should be plural. | Edited |
| 23 | How will your study lay the foundation for improving access to surgical care facilities? This seems like a bit of a reach to me. | Thank you, we have now modified this statement. |
|  | Methodology |  |
| 24 | Line 114 survey should be plural since there were multiple surveys completed | Edited |
| 25 | In the survey tool section, what if a mother was not present in a household to answer the survey? Were fathers or other caregivers allowed to answer? | caregivers present in house provided the information. We have now clarified this in the manuscript. |
| 26 | In lines 145-146, I’m confused as to why you mention the 256 items of the WHO tool without describing the tool. Is it similar to the PediPIPES tool? Why did you choose PediPIPES and not the WHO tool? | Edited and revised |
| 27 | Lines 151-152, same comment about the mothers being the only respondents to the survey? Also, if mothers provided info about children under 5, who provided info for children above the age of 5? I see below that the study is in children under 5. This needs to be made clear much earlier in the paper. | Revised. Made it clear now |
| 28 | How was the info recorded electronically? On Excel? REDCap? More details here. | The data was collected through an android based app that was developed in-house.  The application was developed for Android, and an IIS-10 webserver was setup with MySQL database to collect data in the remote areas. APIs were developed on webserver in PHP to send and receive data to and from devices. The purpose of android-based survey application was to assist data collectors in conducting interviews in the remote field areas and capture data in electronic format. The application was used on Samsung tab A7 tablet. To restrict inappropriate access to the application, authorized data collectors were required to authenticate using login credentials that where specifically generate for the purpose of this application on the server. |
| 29 | I find it confusing that you give the numbers of health facilities in Sindh, but then only report on the 39 health facilities in the district. I don’t think you need the numbers of health facilities in Sindh, but rather just the district to keep things clear and simple. | Thanks for this suggestion, we have now revised this accordingly. |
| 30 | What is the difference between a research coordinator and research specialist? If not applicable to this study, you can probably just say two research personnel. | We understand that these details are not needed, hence have revised it |
| 31 | Line 164 – the mention of the photographs seems random and has not been mentioned elsewhere. What was this for? How does this relate to the study? | A total of 233 photographs were taken of children with lesions. These were reviewed by the relevant surgeons at the Aga Khan University Hospital for diagnosis, and confirmation of the previously established diagnosis by the doctors in TMK. These pictures were taken via the same application developed by the DMU and stored on the tablet/phone under password protection. |
| 32 | Data analysis section, lines 168-169 – when you say current surgical need was self-reported, who is self-reporting this? What interview? With families or health facilities? | Caregivers reported surgical problems in their children, either present at the time of the surveys, or in the past. Interview of the caregiver with our research associate to ask questions about the survey. We have clarified this |
|  | Results |  |
| 33 | In the first paragraph, you summarize the demographics, but do not mention all the demographics. I found myself wondering where the demographics table was. Is there a reason you do not have a demographics table? I would consider adding one. | Demographics information available in table 1 |
| 34 | You do not need to say “a rural district in Pakistan” in line 174 as this has been well described above. | Edited |
| 35 | You included quite a bit of information about deaths, including a lot of info about deaths that are not surgical or related to surgical care. This manuscript is incredibly long, and this could be one way you could shorten it, but removing a lot of the non-surgical related data about deaths. | Thanks, Revised to only surgical causes |
| 36 | In the description of the lesions, you report a lot of the same data both in text and in table form. I recommend cutting out a lot of repetition with having only the highly pertinent data (highest percentages, etc) in the text and then having all the data reported in the tables (like you do currently). As it is now, I am getting lost in the data and unable to make any meaningful conclusions. Hopefully by cutting down the long lists of percentages and frequencies in the text you will be able to only report the high yield results you want readers to gain from this section. | Thanks for this important suggestion, we have now edited all the results for clarity. |
| 37 | Line 271 Photographs – Again, I’m unclear as to how the photographs relate to this study. I’m not sure I would include them as I don’t think they add to your study. I would remove them. | Revised to add pertinent details as to why photographs were taken. |
| 38 | Health seeking behavior section – line 282 please say treatment consisted of mainly medical management, as it was not only medical management. | Thanks. Revised |
| 39 | Line 289 – “children reported”, so children filled out the surgeries? Above you say parents filled them out for children under 5. Were these children over 5? This needs to be clarified as it’s unclear the age of the population and who is filling out each survey. | Revised. Caregivers reported these conditions, only children under five were part of the survey. |
| 40 | Line 291 – children are reporting working? How old are these children? | Clarified the term ‘work’: being able to carry out their tasks the way they were previously able to |
| 41 | Table 4 – please review the capitalization and punctuation in this table and fix errors. An example is that “lesion” is not capitalized and “Health” is in the middle of a sentence. Please do this for all tables. | Fixed all the tables. |
| 42 | Personnel – what personnel were present at the other hospitals, not just the DHQ? | Medical facilities were surveyed to identify presence of trained personnel to handle surgical care which included, pediatric and general surgeon, anesthesiologists, medical doctors, pediatricians, pediatric trained nurses and nurse anesthetics. Only one facility out of the 39 surveyed was identified to have trained personnel present, which comprised of three anesthesiologists, two pediatricians, two pediatric trained nurses and one general surgeon |
| 43 | Infrastructure – this paragraph is centered and looks different than the others. Please fix the formatting here. | Edited |
| 44 | Table 7 and 8 – it would be helpful to list the conditions with greatest frequency/percentage at the top and then with decreasing frequency. I think this would make the table easier to read. Also, the title should be “Equipment”. | Thanks, we have now made this in order of highest to the lowest frequency |
| 45 | Equipment and Supplies – I find the listing of equipment to be a little repetitive and hard to read. You don’t need to list everything that is already present in the table. Just point out relevant results that you want readers to take away as important | Agreed, we have now removed the unnecessary details. |
| 46 | PediPIPES scores – this section needs some major editing because I don’t know what a score of 8, 9, or 12 means. Listing numbers without telling us what they mean is useless. Also, which hospital had the highest scores that you are referring to? What scores did the other hospitals have? It’s hard to know what this section means without details and knowing what the scale of the scores are. | Updated methodology:  PediPIPES personnel, infrastructure, procedures, equipment, and supplies score were calculated by allocating 1 point for each personnel, infrastructure, procedures, equipment and supplies present in the facility. A total PediPIPES score is calculated by summing the number of the Personnel, Infrastructure items and number of operating rooms, Procedure, Equipment and Supplies scores. This number was then divided by the total number of data items (118) and multiplied by 10 to create the PediPIPES index. There is no maximum number for the PediPIPES index.  Result section:  The hospital with the highest PediPIPES index of 5.7 in TMK had the following: Personnel score 8, infrastructure score 9, procedure score 12, equipment score 17 and supplies score of 21. The hospital with the lowest index of 2.4 had the following: Personnel score of zero, infrastructure score of 3, procedure score of 3, equipment score of 9 and supply score of 13. The mean PediPIPES index was found to be of 3.0 in the 39 facilities surveyed. |
|  | Discussion |  |
| 47 | The first two sentences of the discussion would belong better in the introduction as they give good info on the SOSAS survey and why you chose to use it. | Incorporated in the introduction |
| 48 | Lines 360-362 – this sentence about the primary aim of the study is repetitive with the primary aim in the intro. I would rephrase here. | Revised |
| 49 | Lines 363-372 are a restatement of the results rather than summarizing the findings and discussing the findings. You are reporting your results section again here rather than comparing and contrasting with the existing literature. | Revised |
| 50 | Again, lines 373-378 are a restatement of the results. This nicely distills the results and you could almost move this to the results section as I like it better than the long list of findings you currently have in the results, but this does not belong in the discussion. You need to discuss the findings, not restate them in the discussion section | Revised. |
| 51 | In line 382, you say compared to other LMICS, but don’t give the rates of surgical need in other LMICs. Report these rates, and compare and contrast them with your findings. I need more discussion of the existing literature in the discussion section. More like you currently have in lines 386-388! | Revised with comparisons. |
| 52 | The paragraph from lines 389-405 is excellent and has some great suggestions for improvement. I want more of this! | Thanks, revised |
| 53 | Were there any limitations to the health facility assessment part of this study? Who were performing the assessments of the health facilities? Is it possible there could have been errors in reporting capacity? | Trained research personnel from our core staff conducted these assessments. These were well acquainted with the health facilities of the region and have been working in collaboration with these health facilities for a long time. So minimal chances. |
| 54 | Lines 417-420 – the photographs are mentioned here again, but not elsewhere in the discussion. I still feel that removing the photographs is the right choice from this paper. | Revised and justification provided |
| 55 | I think that a stronger closing is needed. What are the next steps, and how does this study lead to them? I would close with that rather than a random comment about incorporating a physical examination. | Revised discussion. |
|  | Reviewer #2: |  |
| 56 | What was the reason behind selection of District TMK? How is this representative of the rural areas of Pakistan? | TMK district was chosen as a site for our project due to our established office in the district, and the availability of manpower and other resources to conduct this large-scale survey. And provided TMK is representative of any rural district of the province Sindh. |
| 57 | Are the results generalizable to other parts of the country/ region? | They are mostly generalizable to any rural areas of Sindh, and to a large degree to rural areas overall in Pakistan. |
| 58 | Instruments: the authors mention that these instruments are tested for the first time in Pakistan- were they tested for validity, inter-rater reliability? | These are validated tools and have been used in various LMICs, but we did test the translations and pilot tested all the tools before starting the actual survey. |
| 59 | This is a purely descriptive study with frequency table of presumed surgical conditions in pediatric population and availability of surgical care in the district. PIPES survey results demonstrate a general lack of access and availability of surgical services, referral system and questionable quality of available services. How these results could be used to a) quantify the magnitude and epidemiology of pediatric surgical conditions requiring surgical intervention or close follow up; b) informing decision makers about upgrading or expanding the services (specifically for pediatric surgery) ; c) support local communities in accessing and receiving appropriate surgical care and curative services; d) health system strengthening- also in terms of task shifting, referrals and infrastructure | Thanks for these valuable suggestions. We have now revised the conclusion of the manuscript to elaborate on these aspects. |
| 60 | It is better not to use abbreviations in the abstract for the first time. | Revised |
|  | Reviewer #3: |  |
|  | Abstract |  |
| 61 | Page 4 ln 23/24 – needs some copyediting. Again, the justification for this study is not well presented here under abstract. The author can make it clearer by rephrasing. | Edited for clarity, and justification. |
| 62 | Pg 4 ln 26/27; the author used least developed country and LMICs interchangeably. I advise the use of LMICs more so that Pakistan falls within this category according to world bank ranking. | Revised the word to LMIC |
| 63 | The abstract did not state any findings on one of the main objectives of this study – health seeking behaviors. | Thanks for pointing this out. We have now revised and added |
| 64 | Also, there is lack of consistency in using abbreviations and definition of abbreviations at first mention. For instance, whereas ERC, NBC, and AKU were defined earlier in abstract, others such as DALY, SOSAS and PediPIPES were only defined in the main body of the article. | Revised |
|  | Introduction |  |
| 65 | Except for copyediting in ln 65/66, the claims and objectives were well explained and contextualised. The conceptualisation of this idea is also novel. I agree with the authors that community assessment of surgical burden of disease in LMICs is critical to understanding the true picture of surgical needs to advocate for policy change or formulation. |  |
|  | Methodology |  |
| 66 | The authors can make the methodology more comprehensive. For example, there is not mention on when and how literature was searched, and on what data bases was the search conducted- albeit this is a primary study, it is still essential to let the readers know how you generated evidence from literature to justify this study. Again, if the literature search was done in 2019/2020 as at when the study was conducted, then, could new evidence have emerged | Several studies have been published describing childhood surgical emergencies in Pakistan. However, through a literature review, it was identified that no community-based survey tool has yet been employed to determine Pakistan’s pediatric surgical disease burden in rural areas (19-21). We also did a recent check. |
| 67 | I am also curious how the authors decided on conducting this research in TMK despite many other provinces in Sindhi of Pakistan. Was this systematically arrived at possibly through a scientific sampling technique? If yes, this can be explained further, please. Authors may wish to check this link for guidance on sampling technique <https://www.ncbi.nlm.nih.gov/pmc/articles/PMC5325924/> | TMK district was chosen as a site for our project due to our established office in the district, and the availability of skilled manpower and other resources to conduct large-scale survey. And also that TMK is representative of any rural district of Sindh and this has been shown in many other surveys that have been done for other childhood illnesses and also socio-economic |
| 68 | Further, I believe the authors can provide detailed information on the choice of only including under-5 children as study population despite the title and research justification suggesting paediatric population as a whole. In other words, a short paragraph to explain inclusion and exclusion criteria will be helpful. Again, any reasons why only mothers were interviewed instead of any head of household or guardian? | More global attention is focused on infectious diseases related to morbidity and mortality in children under five, hence we wanted to explore the factor of surgical disease burden in this age group. We did this initially but have now also completed a large survey of surgical conditions of children in the two -provinces of Sindh and Punjab, we shall be able to publish that soon. |
| 69 | One may require that more information should be provided on the type of electronic method the data was collected onto – google form or Microsoft or excel etc | The application was developed for Android, and an IIS-10 webserver was setup with MySQL database to collect data in the remote areas. APIs were developed on webserver in PHP to send and receive data to and from devices. The purpose of android-based survey application was to assist data collectors in conducting interviews in the remote field areas and capture data in electronic format. The application was used on Samsung tab A7 tablet. To restrict inappropriate access to the application, authorized data collectors were required to authenticate using login credentials that where specifically generate for the purpose of this application on the server. |
| 70 | Kindly consider adding the common language of communication in TMK under ‘location’ subsection. Were these research associates trained in Sindhi given that the questionnaire was translated to this language? | Mentioned. The data collectors were from the same district and spoke Sindhi language. |
|  | Results |  |
| 71 | Tables were clearly labelled and presented. I appreciated the subcategorization of results on surgical needs. All tables were quite informative immediately. | Thank you for the appreciation |
|  | Discussion |  |
| 72 | The authors could be more critical in exploring why despite going to the hospital, children were unattended to, accounting for high mortality. Is it lack of manpower or lack of supplies or infrastructure (ln 364-369)? | A mixture of both. But mainly the lack of availability of trained personnel in the health facilities that were available. |
| 73 | Ln 389-391 is assertive and would need reference(s). Kindly consider. | Referenced |
| 74 | As part of limitation, the authors may also wish to consider if this sample size is adequate to generalise the evidence so generated from this study to Pakistan as a whole. | Added |
|  | Conclusion |  |
| 75 | This section is entirely missing in the main article. However, from the conclusion in the abstract, I am afraid, it appears quite generic and not entirely supported specifically by data of this study. I advise this should be addressed | Added the section with edits and revisions. |
