# Supplementary material for: Assessment of pediatric surgical needs, health-seeking behaviors, and health systems in a rural district of Pakistan
Source: PLOS Glob Public Health. 2022 Dec 30;2(12):e0000810. doi: 10.1371/journal.pgph.0000810 (PMC10021200; doi:10.1371/journal.pgph.0000810)
Supplement: Attachment — Submitted filename: Response to comments.docx [file pgph.0000810.s003.docx]

**Responses to queries: Assessment of pediatric surgical needs, health-seeking behaviors, and health systems in the rural district of Tando Mohammad Khan Sindh, Pakistan, PGPH-D-22-01041**

| S.No |  |  |
| --- | --- | --- |
| 1 | The use of two different tools and population based survey methods are the strengths of study; however, nothing in the results is a news or a novel information for anyone doing health systems research in Pakistan or similar context. | Yes, agree that using two tools strengthens the manuscript. And these findings at the very minimal level could stimulate discussion around this issue of surgical care which is mostly lacking at the policy level. |
| 2 | If the burden of trauma in under 5 is low, then directing scarce resources in training first responders is a waste. First responder courses may be needed in TMK, but not based on this data. | I would like to thank you for outlining this, we have reworded our statement to address this. |
| 3 | I beg to differ from the authors- many countries have spent time, effort, money and capacity development for implementation of WHO trauma guidelines, only to find that random/ patchy implementation does not work until the entire pre-hospital and hospital based trauma system is targeted for improvement. Policy making and unrestricted funding are most important factors in bringing a positive change in trauma care. | Thanks, we have addressed these comments. Thank you for the time, and expert opinion. We really appreciate it. |
